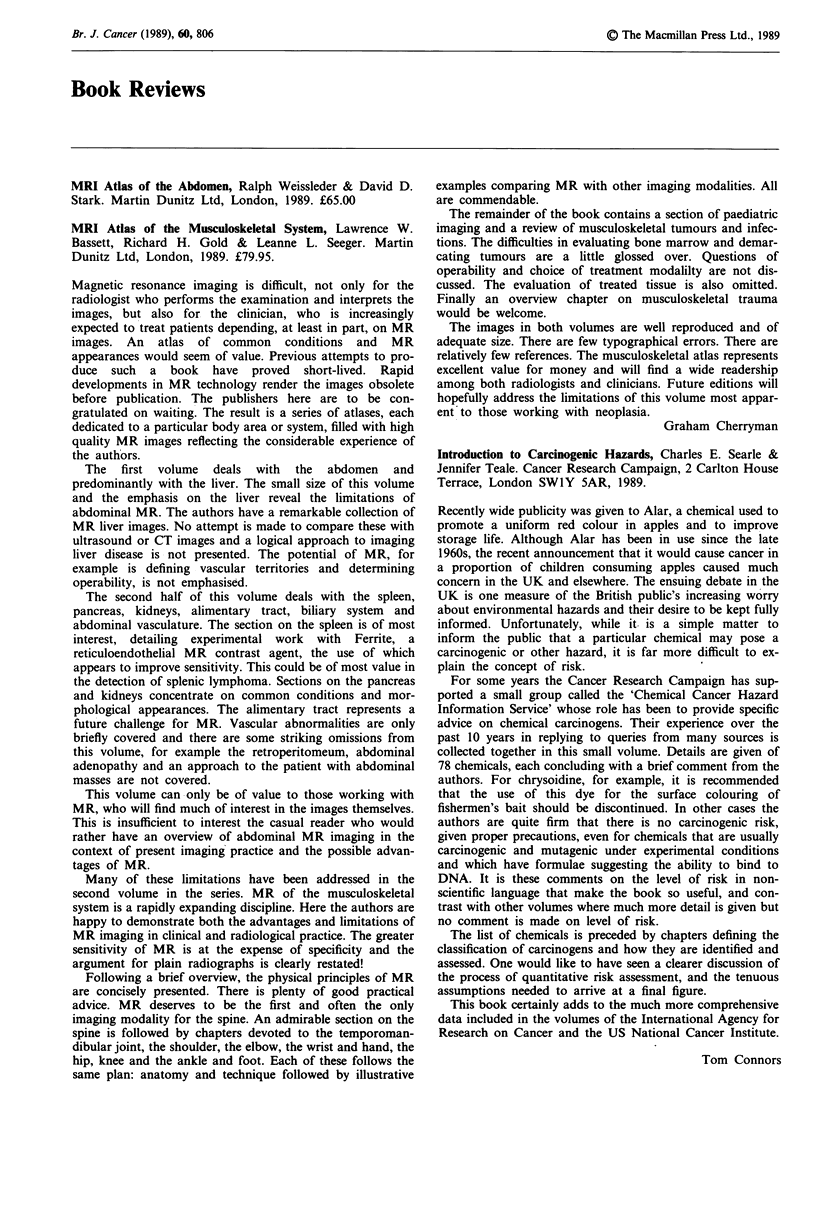# Introduction to Carcinogenic Hazards

**Published:** 1989-11

**Authors:** Tom Connors


**Introduction to Carcinogenic Hazards,** Charles E. Searle & Jennifer Teale. Cancer Research Campaign, 2 Carlton House Terrace, London SW1Y 5AR, 1989.

Recently wide publicity was given to Alar, a chemical used to promote a uniform red colour in apples and to improve storage life. Although Alar has been in use since the late 1960s, the recent announcement that it would cause cancer in a proportion of children consuming apples caused much concern in the UK and elsewhere. The ensuing debate in the UK is one measure of the British public's increasing worry about environmental hazards and their desire to be kept fully informed. Unfortunately, while it is a simple matter to inform the public that a particular chemical may pose a carcinogenic or other hazard, it is far more difficult to explain the concept of risk.

For some years the Cancer Research Campaign has supported a small group called the 'Chemical Cancer Hazard Information Service' whose role has been to provide specific advice on chemical carcinogens. Their experience over the past 10 years in replying to queries from many sources is collected together in this small volume. Details are given of 78 chemicals, each concluding with a brief comment from the authors. For chrysoidine, for example, it is recommended that the use of this dye for the surface colouring of fishermen's bait should be discontinued. In other cases the authors are quite firm that there is no carcinogenic risk, given proper precautions, even for chemicals that are usually carcinogenic and mutagenic under experimental conditions and which have formulae suggesting the ability to bind to DNA. It is these comments on the level of risk in non-scientific language that make the book so useful, and contrast with other volumes where much more detail is given but no comment is made on level of risk.

The list of chemicals is preceded by chapters defining the classification of carcinogens and how they are identified and assessed. One would like to have seen a clearer discussion of the process of quantitative risk assessment, and the tenuous assumptions needed to arrive at a final figure.

This book certainly adds to the much more comprehensive data included in the volumes of the International Agency for Research on Cancer and the US National Cancer Institute.

Tom Connors